# Biodistribution of Quantum Dots-Labelled Halloysite Nanotubes: A *Caenorhabditis elegans* In Vivo Study

**DOI:** 10.3390/ma14195469

**Published:** 2021-09-22

**Authors:** Anna Stavitskaya, Gölnur Fakhrullina, Läysän Nigamatzyanova, Eliza Sitmukhanova, Elnara Khusnetdenova, Rawil Fakhrullin, Vladimir Vinokurov

**Affiliations:** 1 Department of Physical and Colloid Chemistry, Russian State University of Oil and Gas (National Research University), Leninsky Prospect, 65, 119991 Moscow, Russia; eliza.sit@mail.ru (E.S.); husnetdenova.1998@mail.ru (E.K.); vinok_ac@mail.ru (V.V.); 2 Institute of Fundamental Medicine and Biology, Kazan Federal University, Kreml urami 18, 420008 Kazan, Republic of Tatarstan, Russia; GIFahrullina@kpfu.ru (G.F.); lyaysan.nigamatzyanova@gmail.com (L.N.)

**Keywords:** *Caenorhabditis elegans*, quantum dots, in vivo imaging, reproductive toxicity, halloysite

## Abstract

Halloysite is a promising building block in nanoarchitectonics of functional materials, especially in the development of novel biomaterials and smart coatings. Understanding the behavior of materials produced using halloysite nanotubes within living organisms is essential for their safe applications. In this study, quantum dots of different compositions were synthesized on the surface of modified clay nanotubes, and the biodistribution of this hybrid material was monitored within *Caenorhabditis elegans* nematodes. The influence of the modification agent as well as the particles’ composition on physicochemical properties of hybrid nanomaterials was investigated. Several microscopy techniques, such as fluorescence and dark-field microscopy, were compared in monitoring the distribution of nanomaterials in nematodes’ organisms. The effects of QDs-halloysite composites on the nematodes’ life cycle were investigated in vivo. Our fluorescent hybrid probes induced no acute toxic effects in model organisms. The stable fluorescence and low toxicity towards the organisms suggest that the proposed synthesis procedure yields safe nanoarchitectonic materials that will be helpful in monitoring the behavior of nanomaterials inside living cells and organisms.

## 1. Introduction

A nanoarchitectonics approach for targeted production of biomaterials, catalysts, membranes, sensors and smart coatings based on halloysite is a fast-developing area of research [1,2,3,4,5]. In biomedical studies, halloysite clay nanotubes have been extensively studied as a component of food packaging materials, tissue engineering scaffolds, drug delivery systems, bone implants, as antibacterial materials and in cosmetics [6,7,8,9,10,11]. Together with organoclay composites, the synthesis of nanoparticles of various shapes, compositions, and functional properties using halloysite clay tubes as a template is exceptionally promising due to a large number of prospective applications.

Scientific and industrial applications motivate the collection of more data on halloysite-based nanomaterials toxicity and behavior within living organisms [12,13,14]. Pristine halloysite nanotubes are known to be biocompatible, which has been demonstrated in various cell cultures and organisms. When other nanoparticles are synthesized or adsorbed on the surface of clay tubes, their toxicity and behavior might be different from that of pristine nanotubes, which was previously reported based on in vitro and in vivo studies [15,16,17,18].

Currently, just a handful of papers report on the cytotoxicity of semiconductor nanoparticles stabilized on halloysite and even less are based on the studies using the clay-derived nanomaterials in vivo [14,19,20]. One of the reasons to investigate these issues is that the monitoring of pristine halloysite or nonfluorescent probes in vivo is difficult due to the low contrast of materials to living tissues. The anchoring of fluorescent quantum dots (QDs) to nanoclay will be helpful to obtain novel insights about cellular uptake and halloysite transportation within the organisms [21]. We have recently demonstrated that quantum dots synthesized in situ on halloysite were more stable fluorescent probes if compared to traditional fluorescence dyes [22,23].

Quantum dots have a great advantage due to composition-dependent optical properties, such as fluorescence emission spectra, quantum yield and stability. Free QDs have been extensively investigated recently. The CdS, ZnS, and CdSe free QDs, showed to be toxic to *Caenorhabditis elegans* [24]. Nanoarchitectonics approach to the design of fluorescent QDs based on clay tubes and their in vivo studies will help to design less toxic fluorescent probes for bioimaging and on-line monitoring of nanomaterials in living organisms.

*C. elegans* is a free-living soil nematode with a body length of 1 mm and a width of 70–90 µm, which is frequently used as a model organism for studying various processes in biology, including energy metabolism, immunity and aging. Reproduction rate, optical transparency of the body, the short life cycle (3 days), the short life span (2–3 weeks), being not expensive, and relatively easy cultivation in a laboratory make this nematode species an ideal model organism [25]. Importantly, the bioinformatic analysis demonstrated that 60–80% of *C. elegans* genes are homologous to human genes [26]. For this reason, these nematodes are widely used to model complex human diseases, including Alzheimer’s disease [27], Parkinson’s disease [28], diabetes mellitus [29], Duchenne muscular dystrophy [30] and cancer [31]. In addition, the free-living *C. elegans* nematodes are considered as an important alternative in vivo model system for laboratory studies of toxic effects and elucidation of the fundamental mechanisms of the formation of toxicity in engineering nanomaterials [32,33,34].

Microscopy imaging can reveal the uptake efficiency, dispersion state and potential cytotoxicity of nanomaterials. Fluorescence imaging using wide-field fluorescence or confocal laser scanning microscopy is among the most commonly used methods for visualization and tracking of nanomaterials, providing dynamic and real-time information of the interactions between nanomaterials and organisms. In this work, quantum dots with different compositions synthesized on the surface of modified halloysite nanotubes were used for monitoring of the distribution of clay inside *C. elegans* nematodes. Their physicochemical properties were investigated in relation to composition and synthesis procedure. The toxicity of this material was studied in vivo. Fluorescent imaging was compared to the enhanced dark-field hyperspectral imaging (EDF-HSI), which combines dark-field microscopy (DEM) and hyperspectral imaging (HSI), to monitor the nanomaterials inside living organisms.

## 2. Materials and Methods

### 2.1. Materials

Halloysite (HNT), f Furan-2-carbaldehyde (C_5_H_4_O_2_), (3-aminopropyl)triethoxysilane (APTES), cadmium nitrate tetrahydrate (Cd(NO_3_)_2_·4H_2_O) and zinc nitrate hexahydrate (Zn(NO_3_)_2_·6H_2_O) were purchased from Sigma-Aldrich (St. Louis, MO, United States), while thioacetamide (TAA), ammonium hydroxide solution (NH_4_OH) and 96% ethanol and hydrazine hydrate (N_2_H_4_) were all purchased from Acros.

### 2.2. Synthesis of Quantum Dots on HNT

#### 2.2.1. HNT Surface Modification with (3-Aminopropyl)triethoxysilane

A total of 0.2 g of APTES was added to a dispersion of 1 g of HNT in ethanol, and the mixture was kept in an ultrasonic bath for 30 min and then transferred to a closed flask and stirred at 60 °C for 24 h. Afterward, the dispersion was washed several times with ethanol to eliminate an excess of the reagents. The precipitate was dried at 60 °C for 12 h.

#### 2.2.2. Halloysite Surface Modification with 1,2-Bis(2-furylmethyl-ene)hydrazine

A total of 1 g of halloysite was dispersed in 20 mL of hydrazine hydrate and stirred for 30 min. The excess hydrazine was removed by centrifugation. To the precipitate, 30 mL of furfural solution in ethanol was added, and the dispersion was ultrasonicated for 30 min to result in 1,2-Bis(2-furylmethyl-ene)hydrazine (azine) on the surface of clay nanotubes.

#### 2.2.3. QDs Synthesis on the Surface of Modified Clay Nanotubes

**Figure 1:**
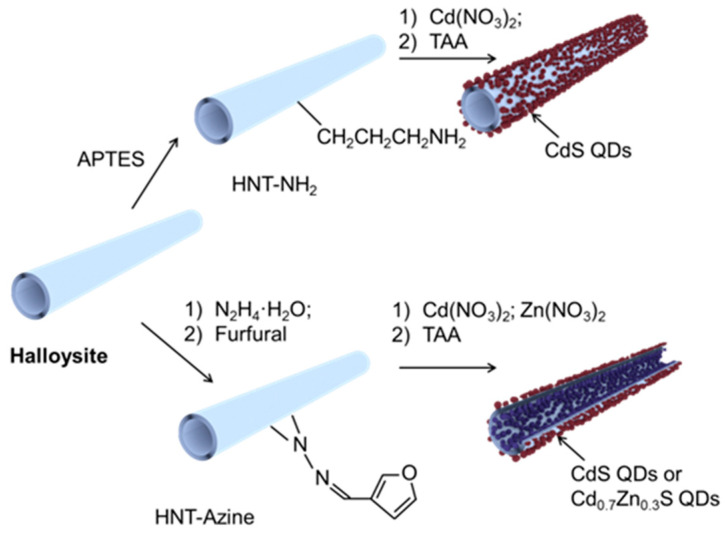
Scheme synthesis of CdS/HNT-Azine, Cd_0.7_Zn_0.3_S/HNT-Azine and CdS/HNT-NH_2_.

For the synthesis of CdS or Cd_0.7_Zn_0.3_S QDs on the surface of clay tubes, the obtained HNT-NH_2_ or HNT-Azine was dispersed in a Cd(NO_3_)_2_ or Cd(NO_3_)_2_/Zn(NO_3_)_2_ ethanolic solution of calculated concentration and kept in an ultrasonic bath for 30 min. Then the TAA ethanolic solution with concentration calculated, taking into account a S/Me molar ratio of 1, was added, and the pH was adjusted to 10 with NH_4_OH. The reaction time was 5 min. After the reaction, the precipitate was centrifuged, washed several times with ethanol and dried at 60 °C for 24 h. The resulting samples were named HNT-NH_2_-CdS, HNT-Azine-CdS or HNT-Azine-Cd_0.7_Zn_0.3_S. The scheme of synthesis is shown in Figure 1.

### 2.3. Nanomaterials Characterization

The morphology of the nanomaterials was studied using transmission electron microscopy (TEM) (JEM-2100, JEOL, Tokyo, Japan). To analyze the elemental compositions of the samples, inductively coupled plasma mass spectrometry (ICP-MS) was used (Agilent, Santa Clara, California, USA). The crystalline structure was analyzed with X-ray phase analysis (Bruker D8, Bruker, Billerica, Massachusetts, USA). The analysis was carried out using Cu Kα radiation (wavelength 0.154 nm, voltage 40 kV, current 40 mA). Spectral characterization was carried out with UV-Vis diffuse reflectance spectra analysis in the range of 350–800 nm with 1 nm of resolution (Jasco V-770, Shimadzu, Tokyo, Japan). The fluorescence spectra of nanomaterials were measured from a solid sample using a fluorometer (Agilent Cary Eclipse, Santa Clara, California, USA) at room temperature in a region from 200 to 600 nm. Zeta-potential measurements were conducted using nanoparticles size analyzer SZ-100 (Horiba, Tokyo, Japan).

### 2.4. In Vivo Studies

Nematode cultures [15] were grown at 20 °C in Petri dishes filled with Nematode Growth Media supplemented with *Escherichia coli* OP50 bacteria as a food source. The sterile eggs were inoculated to the nutrient agar media, and the animals were cultivated to reach the adult hermaphrodite developmental stage. To consider the efficiency of the delivery of nanoparticles and their toxicity, we evaluated certain physiological parameters, such as body length and reproductive potential (egg number per animal). Both body length and fertility were determined using optical microscopy images of adult nematodes at certain times, as indicated. An Olympus BX51optical microscope equipped with CytoViva dark-field condenser was used to study the distribution of nanoparticles in nematodes. Optical bright-field and epifluorescence microscopy experiments were conducted using a Carl Zeiss Imager Z2 upright optical microscope equipped with apochromatic objectives (40×, 63× and 100×). Fluorescence imaging was performed using Carl Zeiss HXP 120 C excitation light source and Carl Zeiss fluorescence FITC narrow band cube (excitation: 475/40 nm, emission: 530/50 nm). Images were captured using an HRC CCD camera (Carl Zeiss). Nematodes were cultivated for 3 days on 6 lunar planets in a solid medium with the addition of the various studied samples of nanotubes (CdS/HNT-Azine, Cd_0.7_Zn_0.3_S/HNT-Azine, CdS/HNT-NH_2_) at a concentration of 1 mg/mL (0.5 mg/mL). The pristine halloysite nanotubes were used as the reference sample.

## 3. Results and Discussion

The spectral characteristics of quantum dots are outlined by their composition and structure; therefore, nanoarchitectonics of semiconductors and semiconductors-based hybrid materials is important for research and industrial applications. Currently, the synthesis of QDs of different compositions in situ on the surface of inorganic nanomaterials and investigation of their properties, including in vivo studies, is poorly studied. Herein, based on the QDs-halloysite composites, we show a novel approach for the synthesis of nanostructured hybrid photonic materials and potential applications of such probes in monitoring nanomaterials in vivo.

The synthesis of CdS/HNT-Azine, Cd_0.7_Zn_0.3_S/HNT-Azine and CdS/HNT-NH_2_ was performed according to a procedure described in [22] (Figure 1). The stabilization of QDs on the surface of clay tubes was achieved by prior surface modification with (3-aminopropyl)triethoxysilane (APTES) or 1,2-Bis(2-furylmethyl-ene)hydrazine (azine). CdS and Cd_0.7_Zn_0.3_S were synthesized in situ on the surface of modified clay following a simple precipitation method. We demonstrate that this kind of synthesis procedure led to the self-organization of sulfide particles with less than 10 nm size selectively on the surface of the clay and not in the bulk. Such materials are quite stable and have good water dispersibility.

**Figure 2:**
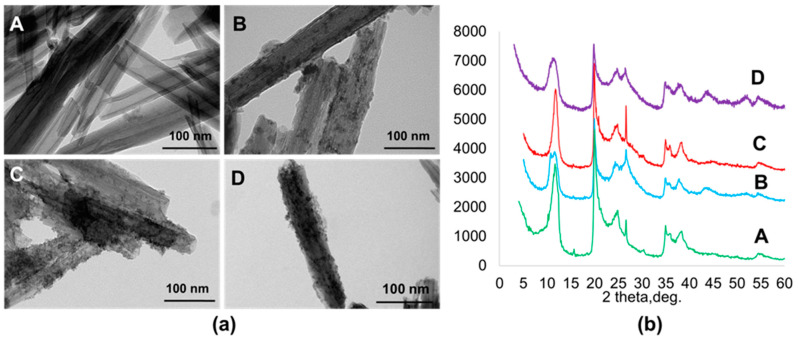
(**a**) TEM image of: A, pristine halloysite; B, CdS/HNT-Azine; C, Cd_0.7_Zn_0.3_S/HNT-Azine; D, CdS/HNT-NH_2_; (**b**) XRD patterns of: A, pristine halloysite; B, CdS/HNT-Azine; C, Cd_0.7_Zn_0.3_S/HNT-Azine; D, CdS/HNT-NH_2_.

TEM investigation revealed that all samples exhibited a homogeneous distribution of QDs on the surface of modified clay tubes (Figure 2a). Due to the close packing of nanoparticles and their transparency under the electron beam, it was difficult to determine particles size distribution using TEM. The crystal structure of materials was confirmed by XRD (Figure 2b). Pristine halloysite displayed characteristic Bragg peaks at 2θ = 11.52, 19.95, 24.63, 34.91 and 54.51 ° [1]. The XRD patterns of functional materials proved the formation of sulfide particles with less than 10 nm (QDs). In the case of halloysite with CdS nanoparticles synthesized on the surface of azine modified halloysite CdS/HNT-Azine, new peaks were revealed at 2θ = 43.01 and 52.54°, corresponding to cubic CdS [35]. For the Cd_0.7_Zn_0.3_S/HNT-Azine, the reflectance peak correspondent to (110) lattice plane shifted to the higher diffraction angles (44.8°), indicating the formation of Cd_0.7_Zn_0.3_S solid solution [36]. CdS/HNT-NH_2_ refractogram had Bragg peaks at 2θ = 43.21 and 52.34°, corresponding to cubic CdS lattice.

Elemental composition analysis was performed using ICP-MS. Cd content was found to be 3.5, 3.2, and 3.9 wt.% in CdS/HNT-Azine, Cd_0.7_Zn_0.3_S/HNT-Azine, and CdS/HNT-NH_2_, respectively. Zn content was 0.9 wt.% in Cd_0.7_Zn_0.3_S/HNT-Azine. This is in good correlation with the calculated amounts of metals used during the synthesis procedure.

Zeta-potential measurements were performed at each reaction stage. We found that successful surface modification with APTES changed zeta-potential for pristine halloysite from −45 to +12 mV. Modification with azine resulted in a slight change in this value to −25 mV. HNTs-QDs composites were characterized by zeta-potential values of −30, −32, and −20 mV in CdS/HNT-Azine, Cd_0.7_Zn_0.3_S/HNT-Azine, and CdS/HNT-NH_2._

**Figure 3:**
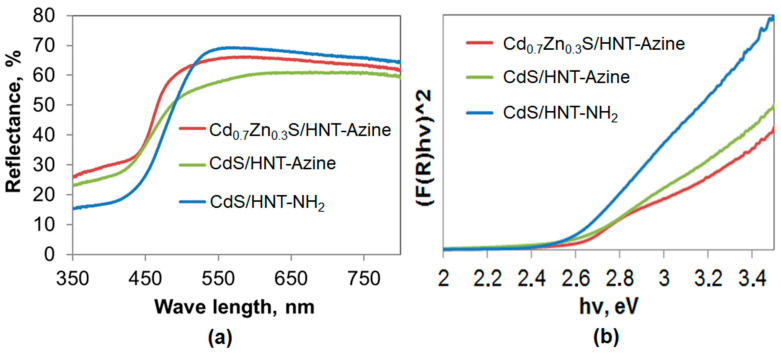
(**a**) UV-Vis diffuse reflectance spectra of CdS/HNT-Azine, Cd_0.7_Zn_0.3_S/HNT-Azine, and CdS/HNT-NH_2_; (**b**) The Tauc plot for CdS/HNT-Azine, Cd_0.7_Zn_0.3_S/HNT-Azine, and CdS/HNT-NH_2_.

Spectral characteristics of QDs-halloysite composite were analyzed to investigate the influence of the composition to the properties of QDs stabilized on clay tubes. A broad absorption region below 500 nm in all samples was attributed to the charge transfer from the valence to the conduction band of QDs (Figure 3a). UV-Vis diffuse reflectance spectra mathematical processing revealed that the band gap of the materials was dependent on the composition of quantum dots (Figure 3b). It equaled 2.52, 2.50, and 2.61 eV for CdS/HNT-NH_2_, CdS/HNT-Azine, and Cd_0.7_Zn_0.3_S/HNT-Azine, respectively. The band gap of a bulk CdS is 2.4 eV [37]. A slight increase in band gap values in the case of CdS stabilized on modified clay could be due to the very small particles size of sulfide crystals. Further increase in the band gap in the case of Cd_0.7_Zn_0.3_S/HNT-Azine was due to the formation of solid Cd_0.7_Zn_0.3_S solution. The addition of Zn in various concentrations increases a band gap of mixed sulfide [38].

**Figure 4:**
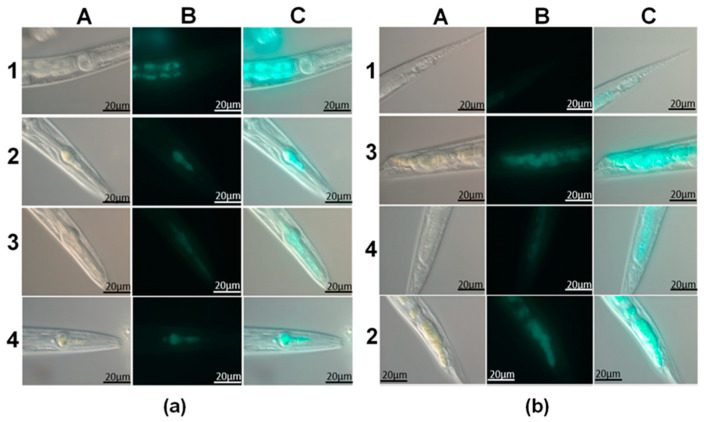
(**a**) Images of the throat of the nematodes fed with fluorescent halloysite nanotubes; (**b**) Tails of the nematodes fed with fluorescent halloysite nanotube. A—optical images, B—fluorescence image, C—alignment of optical and fluorescence images, 1—pristine halloysite, 2—CdS/HNT-Azine, 1—Cd_0.7_Zn_0.3_S/HNT-Azine, 3—CdS/HNT-NH_2_.

In vivo studies of QDs-halloysite composites were performed using *C. elegans* as a model organism. First, we investigated the biodistribution of the clay nanotubes in the nematodes, as shown in Figure 4; in all experiments, halloysite nanotube composites in the form of yellow spots were exclusively seen in the worm’s digestive system, from the bulbus to the anus, with significant congestion in the pharynx, mainly in the extensions, and tail. Nanotubes were also clearly visible in the middle part of the gut, but fewer aggregations were observed. Single isolated halloysite nanotubes were not static or intestine-attached; instead, Brownian motion patterns of halloysite in the intestines of nematodes were detected. It is important to note that halloysite nanotubes were not found outside the nematode intestine. Previous studies indicate that silicon oxide nanoparticles enter the organism of *C. elegans* not only through the oral apparatus but also through the vulva, from where they diffuse into other organs [24].

The distribution of quantum dots stabilized on the surface of halloysite nanotubes inside the intestine of nematodes is shown in fluorescence photographs as bright blue clusters in the pharynx and tail (Figure 4b). In the control experiments, the nanotubes do not show fluoresce; however, the autofluorescence of the lipofuscin droplets is observed in the intestinal cells (Figure 4a(B1)). During the ingestion of bacterial food, nanotubes were pushed down the digestive tract and taken out within an hour. Consequently, nematodes have normal feeding behavior, just like the worms that were fed normal bacterial food (with no nanotubes added). This indicates that the exposure and the need for nanotubes with stabilized quantum dots do not cause a gross behavioral defect in the organism.

**Figure 5:**
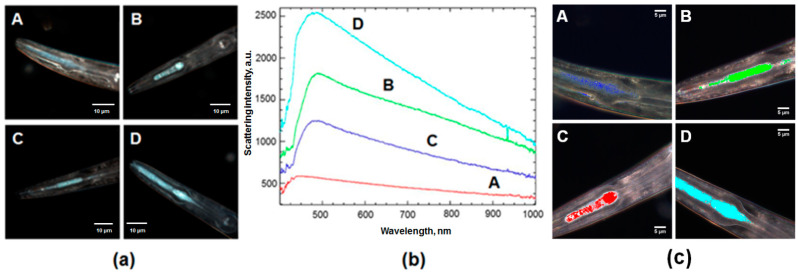
(**a**) Dark-field images and (**b**) the corresponding spectral curves of nematodes feed with: A—pristine halloysite, B—CdS/HNT-Azine, C—Cd_0.7_Zn_0.3_S/HNT-Azine, D—CdS/HNT-NH_2_, (**c**) Hyperspectral mapping of nematodes feed with: A—pristine halloysite, B—CdS/HNT-Azine, C—Cd_0.7_Zn_0.3_S/HNT-Azine, D—CdS/HNT-NH_2_.

The dark-field images (Figure 5a) confirm the effectiveness of uptake of halloysite nanotube decorated with stabilized quantum dots on their surface. Apart from dark-field images, the light scattering intensity of nanomaterials were studied in the hyperspectral microscopy mode. The corresponding spectra (absorption peak ranges) differ from those of the initial halloysite nanotubes and from each other due to QDs composition and surrounding differences (Figure 5b,c). The most intensive signal was obtained in the case of QDs supported on halloysite modified in APTES. This is more likely to be attributed to surface modification. Earlier it has been shown that the optical scattering intensity of the peptide nanoparticles was enhanced by the amidation [39]. Figure 3a also confirms the higher light reflection intensity of this material. As for azine modified halloysite with Cd_0.7_Zn_0.3_S, it has a higher light adsorption. Halloysite UV-Vis diffuse reflectance analysis showed that it reflects more than 80% of light in UV and visible regions [40]. Despite this, hyperspectral microscopy showed that its modification leads to better image qualities.

**Figure 6:**
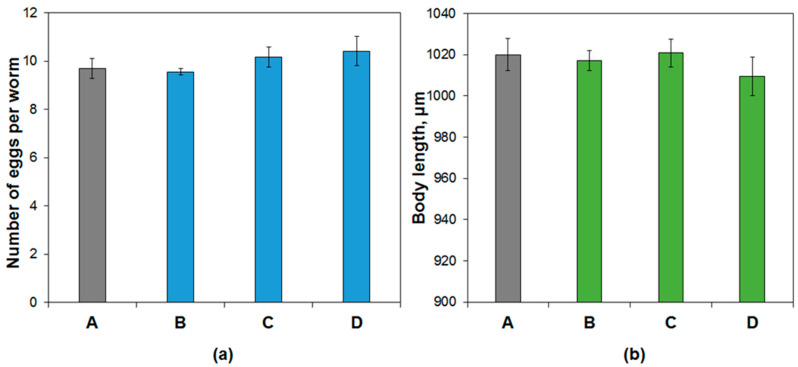
(**a**) The reproductive ability and (**b**) the body length of *Caenorhabditis elegans* after incubation with: A—pristine halloysite, B—CdS/HNT-Azine, C—Cd_0.7_Zn_0.3_S/HNT-Azine, D—CdS/HNT-NH_2_.

We then investigated some physiological parameters in nematodes exposed to CdS/HNT. We found that long-term exposure (from stage L1 to an adult) with the nanotubes does not cause abnormalities in the reproductive organs of nematodes associated with the appearance of the BOW phenotype, in which fertilized embryos hatch inside the mother’s body and begin to feed on its tissues (Figure 6). In the case of free QDs, the short (12 h to 3 days) and especially long-term (16 days) exposure leads to the accumulation of QDs in the uterus and vulva, the eggs were full of QDs even after short term exposure [26]. Difficulty in laying the eggs, damaged eggs left in the vulva, and damaged eggs without an intact eggshell were observed in this study after 1 day of exposure. As can be seen from Figure 6a, the synthesized samples of HNT-NH_2_-CdS, HNT-Azine-CdS, and HNT-Azine-Cd_0.7_Zn_0.3_S did not affect the reproductive ability of nematodes. By comparing the growth in body length of *C. elegans* nematodes exposed to CT, we can determine the sensitivity to toxins and toxicity at an early stage of exposure. As can be seen from Figure 6b, the body length of nematodes developing under the influence of nanomaterials did not change significantly. These facts indicate the absence of any acute toxic effects on model organisms in vivo.

## 4. Conclusions

A new approach that may help in monitoring the behavior of nanomaterials inside living organisms by the anchoring of QDs on their surface was proposed in this study. Fluorescent quantum dots synthesized on the surface of modified clay nanotubes were used to study the distribution of halloysite clay tubes within the body of model nematodes *C. elegans*. It has been shown that using fluorescent probes with stable fluorescence, it is possible to study the distribution of clay tubes that are extensively studied as drug delivery polymer-composite systems, in food polymeric packages, and other polymer materials. This approach may help to obtain new data on such important issues as nanomaterials toxicity, cells uptake, and the traveling of nanoparticles within a body. It has been stated that these fluorescent QDs stabilized on halloysite nanotubes showed no acute toxic effects on reproduction ability, life cycle, and behavior of model nematodes. It is especially worth noting that the nanomaterials were not found in the uterus, spermatheca, and nematode embryos.

## Data Availability

The data presented in this study are available on reasonable request from the corresponding authors.
